# Modifiable risk factors and atypical cognitive impairment relationships among diverse older adults in the NACC dataset

**DOI:** 10.1002/alz70860_101594

**Published:** 2025-12-23

**Authors:** Giovanna Pilonieta

**Affiliations:** ^1^ University of Alabama at Birmingham, Birmingham, AL, USA; Alzheimer's Disease Center, Birmingham, AL, USA

## Abstract

**Background:**

An estimated 40 % of Alzheimer's disease and related dementias may be attributable to modifiable factors. Characterizing modifiable risk factors associated with cognitive impairment and dementia is essential for designing and implementing precision medicine approaches to maintain and minimize cognitive and functional decline for individuals at higher risk and among older adults.

**Method:**

The study used the Uniform Data Set (UDS) of the National Alzheimer's Coordinating Center (NACC) from March 2015 to September 2024 to compare demographics, clinical characteristics, social contact, and cognitive and functional performance variables in adults with baseline cognitive status of impaired‐not‐MCI. Bivariate analyses evaluated differences in modifiable risk factors by race among Non‐Hispanic Black (NHB; *N* = 184; 38.33%) and Non‐Hispanic White participants (NHW; *N* = 296, 61.67%). Spearman's rank correlations examined the associations between CDR^®^, MoCA, MINT, and FAS scores.

**Result:**

Among 480 participants, 62.71% were female, mean age was 68.40 (SD 7.93), and mean education was 15.56 (2.82) years. NHB had a higher proportion of female participants (*χ*2 = 17.6105; < .0001), were younger (t=3.55, 0.0004), had fewer years of education (t=7.32, < .0001), were less likely to report a first‐degree family member with cognitive impairment (*χ*2=50.2889; < .0001) and had lower CDR‐GS (*p* <0.050), MoCA (*p* < .0001) and MINT (< .0001) scores. Regarding modifiable risk factors, NHB were more likely to live alone (*χ*2 = 56.4668, < .0001), be current smokers (*χ*2=32.7631; < .0001), and report hypertension (*χ*2 = 25.4184;< .0001), and diabetes type II (*χ*2=23.7612;< .0001) but less likely to report alcohol intake (*χ*2 = 14.8833; 0.0006). There were significant correlations among the assessments. However, associations between MoCA and FAS scores were only significant among NHB enrollees.

**Conclusion:**

Our study found differences in modifiable risk factors for dementia, and the associations between cognitive and functional assessments by race. Future research should explore longitudinal associations between environmental, social, and individual factors and biomarkers underlying atypical cognitive impairment patterns among diverse older adults.

